# Shear-Thinning Extrudable Hydrogels Based on Star Polypeptides with Antimicrobial Properties

**DOI:** 10.3390/gels10100652

**Published:** 2024-10-11

**Authors:** Dimitrios Skoulas, Muireann Fallon, Katelyn J. Genoud, Fergal J. O’Brien, Deirdre Fitzgerald Hughes, Andreas Heise

**Affiliations:** 1 Department of Chemistry, RCSI University of Medicine and Health Sciences, 123 St. Stephen’s Green, D02 YN77 Dublin, Ireland; andreasheise@rcsi.ie; 2 Department of Clinical Microbiology, Royal College of Surgeons in Ireland, RCSI Education and Research, Beaumont Hospital, Beaumont, D09 V2N0 Dublin, Ireland; muireannfallon@rcsi.ie (M.F.); dfitzgeraldhughes@rcsi.com (D.F.H.); 3 Tissue Engineering Research Group, Department of Anatomy and Regenerative Medicine, RCSI, D02 YN77 Dublin, Ireland; katelynjgenoud@rcsi.ie (K.J.G.); fjobrien@rcsi.com (F.J.O.); 4 AMBER, The SFI Advanced Materials and Bioengineering Research Centre, D02 YN77 Dublin, Ireland; 5 Science Foundation Ireland (SFI) Centre for Research in Medical Devices (CURAM), D02 YN77 Dublin, Ireland

**Keywords:** polypeptides, star polymers, antimicrobial potency, hydrogels

## Abstract

Hydrogels with low toxicity, antimicrobial potency and shear-thinning behavior are promising materials to combat the modern challenges of increased infections. Here, we report on 8-arm star block copolypeptides based on poly(L-lysine), poly(L-tyrosine) and poly(S-benzyl-L-cysteine) blocks. Three star block copolypeptides were synthesized with poly(S-benzyl-L-cysteine) always forming the outer block. The inner block comprised either two individual blocks of poly(L-lysine) and poly(L-tyrosine) or a statistical block copolypeptide from both amino acids. The star block copolypeptides were synthesized by the Ring Opening Polymerization (ROP) of the protected amino acid N-carboxyanhydrides (NCAs), keeping the overall ratio of monomers constant. All star block copolypeptides formed hydrogels and Scanning Electron Microscopy (SEM) confirmed a porous morphology. The investigation of their viscoelastic characteristics, water uptake and syringe extrudability revealed superior properties of the star polypeptide with a statistical inner block of L-lysine and L-tyrosine. Further testing of this sample confirmed no cytotoxicity and demonstrated antimicrobial activity of 1.5-log and 2.6-log reduction in colony-forming units, CFU/mL, against colony-forming reference laboratory strains of Gram-negative *Escherichia coli* and Gram-positive *Staphylococcus aureus*, respectively. The results underline the importance of controlling structural arrangements in polypeptides to optimize their physical and biological properties.

## 1. Introduction

One of the biggest threats to the modern health system is the increasing range of nosocomial infections caused by several opportunistic pathogens [1,2]. Open wounds or cutaneous lesions of the body can quickly become colonized with microbes from the human skin microbiome [3,4]. During the tissue regeneration or wound healing process, the tissue is very susceptible to infections caused by these microbes, which, if unchallenged by a compromised immune response, may become chronic, with life-threatening or life-changing consequences [5]. Although systemic antibiotics and topical antiseptics are used to mitigate these consequences, antimicrobial resistance (AMR) hinders the effective prevention and treatment of infections. An emerging family of materials that show antimicrobial activity that may be clinically applicable in topical antimicrobial management of wound infections are antimicrobial peptides (AMPs). AMPs kill bacteria through mechanisms different to antibiotics [6,7]. AMPs exhibit electrostatic interactions with the membranes of the microorganisms [8], distinguishing them from antibiotics, which target cell wall synthesis, protein synthesis or DNA replication/transcription [9]. However, several disadvantages, such as the high cost of peptide synthesis and the lack of sufficient AMP selectivity between pathogens and mammalian cells, have limited the integration of AMPs into therapeutic materials [10,11]. Thus, the biggest challenge remains the development of biomaterials with antimicrobial properties while maintaining minimum toxicity to healthy tissues. A strategy to overcome these barriers is the development of readily synthesizable polymers with antimicrobial potency [12,13].

Ring Opening Polymerization of N-carboxyanhydrides is a widely used method for synthesizing polypeptides with antimicrobial properties [14,15]. Specifically, polypeptides comprising cationic lysine (Lys) have received attention due to their ability to disrupt microbial membranes [16]. An example is a diblock copolypeptide of the type poly(L-lysine)-*b*-poly(L-phenylalanine) which displayed strong antimicrobial activity [17]. Another parameter that can affect the efficacy of the polypeptides against microbes is their chain structure or molecular architecture. Lam et al. reported on antimicrobial star polypeptides [18], of which the individual polypeptide arms comprise copolymers of cationic lysine (Lys) and hydrophobic valine (Val). It was shown that these polymers exhibit sub-μM activity against all Gram-negative bacteria tested, including a range of antibiotic-resistant pathogens, while retaining low cytotoxicity. Most importantly, antimicrobial resistance was not observed with these materials. Several other examples of antimicrobial star polymers have been reported since, including studies from our own laboratory [19,20,21].

In a separate line of research, our group developed polypeptide hydrogels with advanced properties, some of them suitable for 3D printing [22]. One specific recent goal was to render these hydrogels intrinsically antimicrobial [23]. While there are examples of hydrogels loaded with metal nanoparticles or antibiotics for local administration, these systems rely on the loading and release of antimicrobial agents [24,25,26,27]. In contrast, the integration of antimicrobial polypeptides into hydrogels provides a novel platform in which antimicrobial properties are built into the materials. Antimicrobial hydrogels may provide alternative or complementary topical approaches to the management of some infections [28]. In that context, we recently presented a linear polypeptide design that demonstrates a combination of the above-mentioned features [29]. Based on that, we envisaged that the star architecture could give access to novel materials with superior rheological characteristics, lower toxicity and improved innate antimicrobial properties that could exhibit shape extrudability. In this study, we systematically investigate how the architecture and composition of star-shaped polypeptides influence the properties of hydrogels, including their antimicrobial performance. The overall concept aims to combine several properties in one material—hydrogelation, printability, antimicrobial properties and biocompatibility—to uncover highly potent structures and multifunctional hydrogels.

## 2. Results and Discussion

### 2.1. Design, Synthesis and Characterization of Star Polypeptides

**Figure 1:**
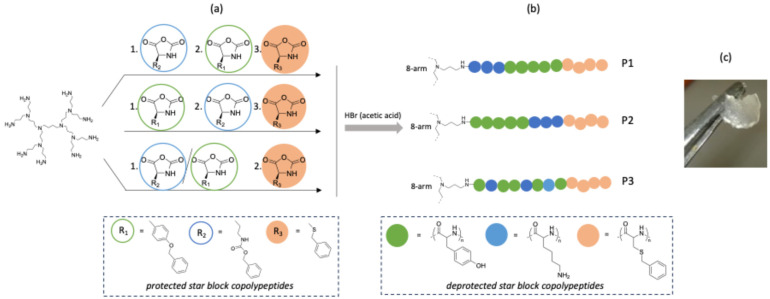
(**a**) Synthesis of the protected star block copolypeptides by NCA ring-opening polymerization from an 8-arm PPI dendrimer; (**b**) structures of the deprotected star block copolypeptides (P1–P3); (**c**) image of the hydrogel obtained from P3.

Figure 1 depicts the design principle followed in this study. A second-generation polypropylene imine (PPI) dendrimer was utilized as an 8-arm initiator for the polymerization of selected amino acid NCAs. We envisaged that the presence of more than one branching point would assist the formation of interconnected networks [30]. The star polypeptides comprised three amino acids selected to add specific functionality to the hydrogel and arranged as polypeptide block structures around the PPI core. PolyLys (pLys) provides hydrophilicity as well as the antimicrobial properties [13,18,31]. *S*-benzyl-*L*-cysteine (pCys(Bz)) is an exceptional hydrogelator as its sheet-like secondary structure stabilizes the hydrogel network [32]. Moreover, it provides hydrophobicity and enhances aromatic interactions as well as hydrogen bonding interactions between polypeptide chains. The addition of this polypeptide block strengthens the β-sheet secondary structure, thereby increasing the hydrogel’s mechanical properties [33]. A tyrosine block (pTyr) can efficiently form non-covalent interactions in water [34]. Furthermore, it promotes the physical interaction within the hydrogel network, as its phenol side groups can form intramolecular hydrogen bonds both with water molecules and with the carbonyl groups of the polypeptide backbone [35]. Additionally, the long arms of the star polymers could promote bacteria membrane depolarization properties [36].

To investigate the impact of the structural arrangement of the amino acids, three different star polypeptides were synthesised by one-pot consecutive polymerization of N(ε)-benzyloxycarbonyl-L-lysine (L-Lys(Z)) NCA, O-benzyl-L-Tyrosine (L-Tyr(Bz)) NCA and S-benzyl-L-cysteine (L-Cys(Bz)) NCA initiated by PPI dendrimer with eight primary amines as initiating points (Figure 1). Two polymers were obtained with three blocks in total and different sequences of the p(Lys) and p(Tyr) inner blocks, namely P1 (PPI-p(Tyr-*b*-Lys-*b*-Cys(Bz))), P2 (PPI-p(Lys-*b*-Tyr-*b*-Cys(Bz))) and a third one with two blocks in total and a statistical inner block of p(Lys) and p(Tyr), namely P3 (PPI-p(Lys-*co*-Tyr-*b*-Cys(Bz))). The p(Cys(Bz) block was always positioned as the outer block of the star polypeptides enhancing the hydrogel’s rigidity [37]. The overall ratio of the three monomers was kept constant with 400 repeating units of Lys, 80 repeating units of Tyr and 80 repeating units of Cys(Bz). The addition of each block was performed after consumption of the NCA(s) of the previous block, as confirmed by measuring aliquots from the polymerization solution using Fourier transform infrared spectroscopy (FT-IR) by testing aliquots from the polymerization solution. The full disappearance of the bands at 1780 cm^−1^ and 1850 cm^−1^, indicative of NCAs, verified the successful polymerization, while the presence of a peak at 1650 cm^−1^ is strong evidence of the polypeptide bond. Reaction aliquots were also monitored by Size-Exclusion Chromatography (SEC). Clear shifts in molecular weights were observed between the first and second inner block of the samples P1 and P2 (Figures S14 and S15 and Table 1). Since the star polypeptides were not soluble in common SEC solvents after the polymerization of the outer p(Cys(Bz)) block, it was not possible to obtain SEC results of the final star block copolypeptide (Figure S13).

**Figure 2:**
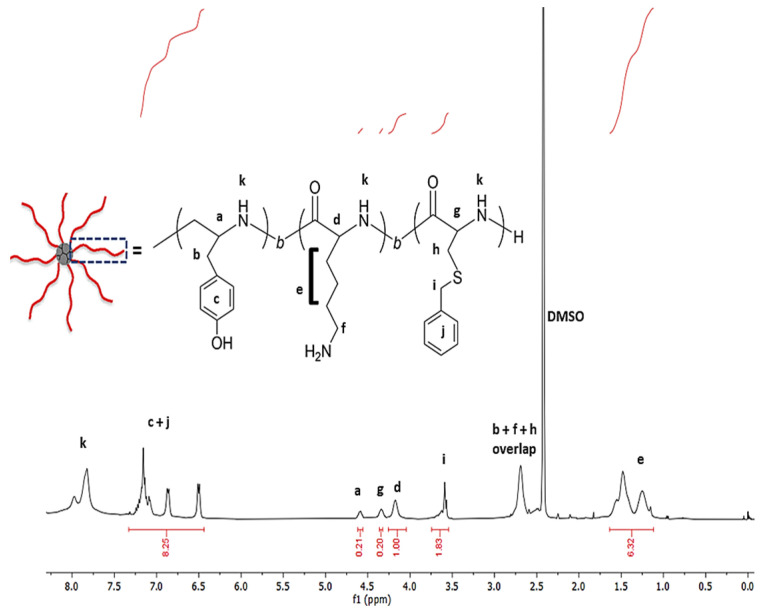
^1^H NMR spectrum of deprotected P1 (DMSO-d6) and schematic polymer structure with signal assignments.

As the final step, the pLys and pTyr blocks were deprotected via acid hydrolysis and characterized by ^1^H NMR (Figure 2). Even though the calculation of the degree of polymerization is not possible due to the absence of distinctive initiator signals, the composition of the monomers Lys, Tyr and Cys(Bz) was found to be largely in agreement with the feed ratio of NCAs. Moreover, ^1^H NMR spectra show the removal of the protective groups of Lys and Tyr (absence of signals at 5.0 ppm), leaving the protecting group of cysteine intact under the applied conditions in agreement with the literature examples, as evident from the signals at 3.65 ppm [32].

### 2.2. Rheological Investigation and Hydrogelation

Addition of deionized water to the three star block copolypetides afforded the hydrogels. Differences in the water uptake of the samples were noted and ascribed to the differences in polypeptide chain structure (Table 2). Of the three samples, hydrogel P3 can take up the highest amount of water. Furthermore, the extrudability through needles of different gauche size was investigated (Table S1). It was found that P3 can be extruded through very thin needles, even the thinnest 27G, which provides the first evidence of shear-thinning behavior. More qualitative data were obtained from the rheological investigation of the hydrogels. To further validate the rheological properties, two different sets of dynamic oscillatory measurements were conducted. First, Dynamic Strain Amplitude Sweeps (DSS) at constant frequency and increasing strain amplitude were performed to identify the linear viscoelastic window. Second, Dynamic Frequency Sweeps (DFS) were performed to probe the rheological behavior of the linear viscoelastic spectrum.

**Figure 3:**
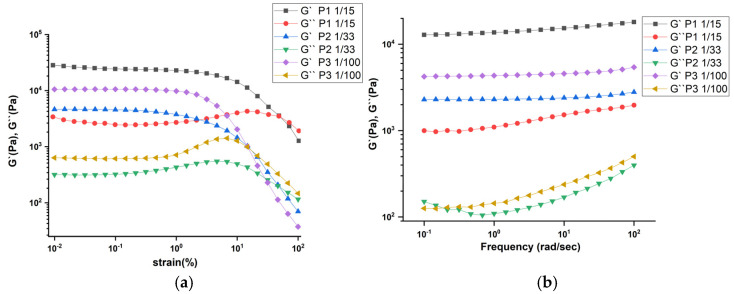
(**a**) Strain-dependent storage G′ and loss G″ moduli for the hydrogels P1, P2 and P3. (**b**) Frequency-dependent storage G′ and loss G″ moduli for the hydrogels P1, P2 and P3.

The dynamic strain amplitude sweep data (Figure 3a) suggest that the hydrogels exhibit a linear viscoelastic regime and a G′ − G″ crossover indicating the transition from a gel-like to a liquid-like regime. This shear-thinning behavior upon application of nonlinear shear results in viscous flow through a syringe needle. Additionally, the hydrogels can self-recover and reshape, facilitating the extrudability of the hydrogels. The viscoelastic properties of P3 can therefore be controlled by the polymer/water ratio (Figure S18). The hydrogels are characterized by a large and extended plateau modulus as suggested by the Dynamic Frequency Sweep data (Figure 3b).

**Figure 4:**
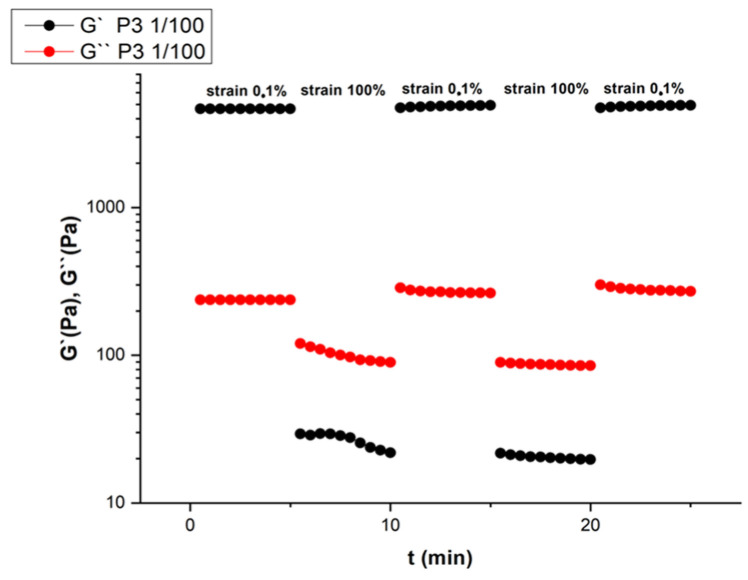
Time-dependent storage G′ and loss G″ moduli of the hydrogel P3.

To examine the rapid self-recovering and self-healing properties of these materials, a time-dependent amplitude sweep cycling test between low strain and high strain was carried out (Figure 4). A rapid decrease of G′ followed by its rapid increase to the initial value was observed by applying a high shear strain followed by a lower strain. In Figure S17, the frequency response in terms of loss factor is presented. The results provide a better understanding of the inverse degree of elasticity (tan(δ) = G″/G′), since the lower its value, the larger the elastic contribution over the viscous one, something very evident for sample P3.

**Figure 5:**
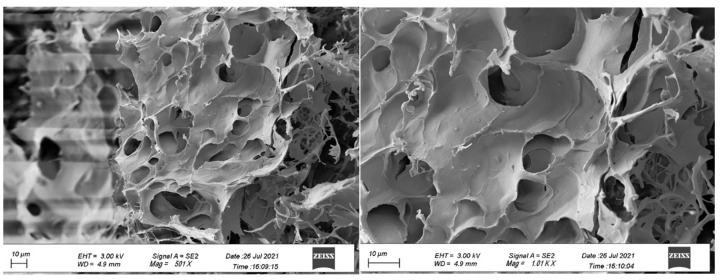
Representative SEM pictures of lyophilized P3 hydrogels.

The difference between the three star block copolypetides is the block structure of the Lys/Tyr inner block(s). While in P1 and P2 these two amino acids are arranged as two individual blocks, in P3 they form a single block with a statistical arrangement of the two monomers. This has a measurable impact on the hydrogel properties; to gain more insights into the underlying reason, secondary structure analysis was carried out. Typically, this can be done by circular dichroism (CD) but the insolubility of P1 and P2 in water prevented their examination. Computing the CD data of P3 by BeStSel software revealed percentages of secondary conformations of 43.4% coil, 41.2% β-sheet and 15.4% β-turn. The data suggest that the P3 sample adopts a mixture of random coil and β-sheet/turn conformation. It is speculated that the β-sheets arise from the outer pCys(Bz) block and are stabilizing the hydrogel. The high content of random coil conformation may arise from the statistical Lys/Tyr inner block giving the molecule a high molecular flexibility. This agrees with the FTIR examination, which reveals β-sheet assemblies (1642–1620 cm^−1^), as well as peaks that support the existence of random coil motifs (1652–1648 cm^−1^). The results clearly highlight the influence of the arrangement of the Lys and Tyr as the inner block on the hydrogel properties. On the molecular level, we hypothesize that a random arrangement facilitates the formation of hydrogen bonds between charged ε-ammonium groups of Lys with uncharged acceptors such as tyrosine [38,39,40,41], thereby enhancing the physical properties of the hydrogel. Compared to a more restricted scenario when both Lys and Tyr are individual blocks, as in P1 and P2, the random arrangement of both monomers, in addition to the hydrophilic–hydrophobic interactions, causes the formation of a stronger hydrogel by P3. Lyophilized samples of the hydrogels were imaged by Scanning Electron Microscopy (SEM). The images show that samples form interconnected membranes and 2D structures with thick sheets and voids with porosity of various sizes (Figure 5). The formation of fibrils is not so intense or evident. It can be perceived that polypeptides form 3D structures with the obvious presence of cavities, a typical feature of physical hydrogels based on polypeptides [42]. No change in the size of the cavities is observed as a result of the different arrangement of the blocks. The same motif is repeated for all the hydrogels.

Overall, the results prove that P3 forms a stronger and more elastic hydrogel; at the same time, it absorbs higher amounts of water and can be extruded through the thinnest needles. As a result, P3 was selected as the lead material for further testing and evaluation at its maximum ratio of polymer mass to hydrogel mass (1/100).

### 2.3. In Vitro Cytotoxicity, Antimicrobial Potency and 3D Printing

**Figure 6:**
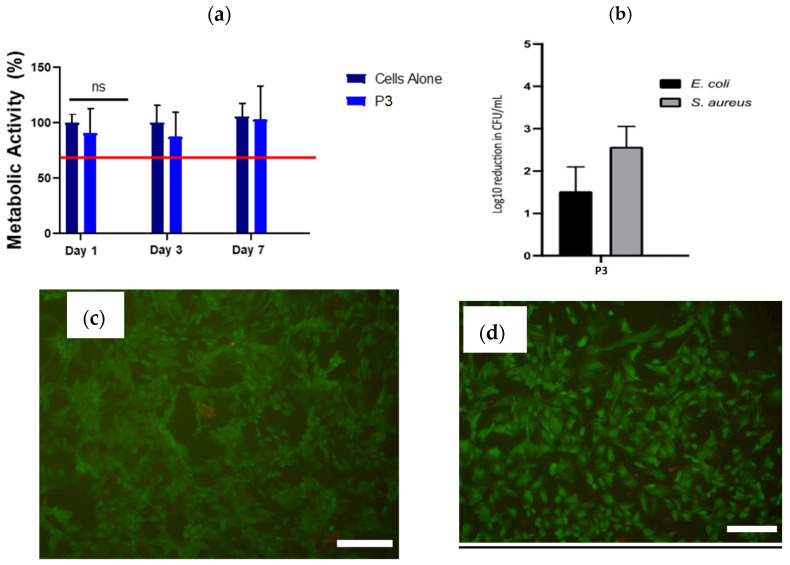
(**a**) Percentage of metabolic activity of rat MSCs (n = 3) over 7 days in culture with hydrogel P3 as determined via alamarBlue© (excitation 545 nm and emission at 590 nm). The red line represents the 70% cut-off for cytocompatibility as per the ISO standard. Metabolic activity is presented as mean ± SEM and compared to that of cells not exposed to hydrogels (cells alone); (**b**) log reduction in colony-forming units (CFU) mL^−1^ following application of approximately 10^5^ CFU/mL *S. aureus* (ATCC25923) or *E. coli* (ATCC25922) to hydrogel P3; (**c**,**d**) representative examples of LIVE/DEAD™ fluorescent imaging of rMSCs exposed to the P3 hydrogel after 7 days (scale bars 200 μm); (**c**) cells not exposed to hydrogel and (**d**) cells exposed P3 hydrogel.

In vitro cytotoxicity investigation was carried out on rat mesenchymal stem cells (rMSCs) to evaluate the potential cytotoxicity of the P3 hydrogel. The hydrogel was placed in a Transwell^®^ insert above the rMSCs and the metabolic activity of the cells at days 1, 3 and 7 compared to untreated cells was measured (Figure 6a). No cytotoxicity to rMSCs in culture due to leachables was evident from P3 hydrogels over 7 days of exposure. These findings are based on cell viability above the 70% threshold recommended by ISO 10993-5 standard. Additionally, LIVE/DEAD™ stained cell images of the rMSCs were taken after 7 days of exposure to the hydrogels (Figure 6c,d). The resulting images show healthy viable cells (green) with few dead cells (red) when treated with P3 hydrogels, similar to untreated cells, which supports the metabolic data presented in Figure 6a.

**Figure 7:**
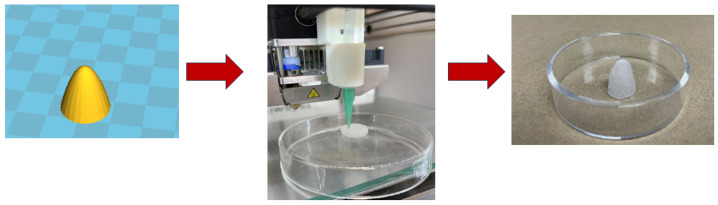
A cone structure showing high control of hydrogel 3D printing from 3D CAD file to hydrogel construct.

Next, antimicrobial tests were performed on a freshly prepared P3 hydrogel against Gram-negative *E. coli* and Gram-positive *S. aureus*. Sterile water was added to the dry polypeptide at the same ratio used in rheology and in vitro toxicity tests. After incubation with *E. coli* or *S. aureus*, the colony-forming units CFU/mL (surviving bacteria) were recorded. Figure 6b shows the inactivation of the two types of bacteria when added to hydrogel P3. Incubation of *S. aureus* and *E. coli* with the hydrogel resulted in 2.6- and 1.5-log reduction in CFU/mL, respectively. The results demonstrate good antimicrobial properties of the P3 hydrogel with no significant cytotoxicity. Finally, P3 was tested for the extrusion printing of simple geometries, like a cone (Figure 7), by extruding through a 23G needle. Printed layers had good melding between them with high shape retention. It was noticed that P3 at lower ratios than 1/100 was not suitable for extrusion printing because water was expelled when it was subjected to shear rate. While extrusion printing of P3 hydrogels would not allow to print more delicate structures, as demonstrated for other hydrogels, it allows simple shapes to be manufactured to match wound geometries.

## 3. Conclusions

Three 8-arm star block copolypeptides based on lysine, tyrosine and S-benzyl cysteine were synthesized with varying block arrangements by ROP of the respective amino acid NCAs. All synthesized polymers spontaneously formed hydrogels in deionized water. Clear differences in their rheological properties and extrudability through different needle gauges were observed as a function of the chain architecture. The polypeptide with a random tyrosine/lysine inner block and S-benzyl cysteine as the outer block displayed favorable viscoelastic properties compared to the triblock copolypeptides, enabling its extrusion through small needle gauges. Moreover, these samples displayed no cytotoxicity and demonstrated antimicrobial. The results highlight the impact of structural features, in this case block arrangement, for otherwise identical polypeptides on their properties. This information is particularly useful in the design of novel antimicrobial polypeptides and can influence future optimization of these materials.

## 4. Materials and Methods

Materials. S-benzyl-L-cysteine and O-benzyl-L-tyrosine were purchased from Bachem. ε-carbobenzyloxy-L-lysine and triphosgene were purchased from Fluorochem, (Derbyshire, UK). All solvents used were anhydrous. PPI (polypropylene imine) dendrimers generation 2 (G2 PPI) was purchased from SyMO-Chem BV (Eindhoven, The Netherlands). α-Pinene, trifluoroacetic acid (TFA), chloroform (CHCl_3_ anhydrous), trifluoroacetic acid-d (TFA-d), ethyl acetate (anhydrous), 1,1,1,3,3,3-hexafluoro-2-propanol (HFiP), tetrahydrofuran (THF anhydrous), hydrogen bromide (HBr) solution 33 wt.% in acetic acid, n-hexane and diethyl ether were supplied by Sigma-Aldrich (Taufkirchen, Germany). Dimethylsulphoxide-d6 (DMSO-d6) was purchased from Apollo Scientific (Bredbury, UK).

Methods and Instrumentation. ^1^H NMR spectra were recorded on a Bruker Avance 400 (400 MHz) spectrometer (Bruker, Ettlingen, Germany). All chemical shifts are reported in parts per million (ppm) with tetramethylsilane (TMS) as an internal reference. Attenuated total reflection (ATR) Fourier transform infrared spectroscopy (FT-IR) measurements were performed on a ThermoFisher Nicolet iS10 instrument. Spectra were obtained from 16 scans with a resolution of 2 cm^−1^ in the spectral region of 500–4000 cm^−1^. Samples were analyzed without prior preparation both in the liquid and solid form. Size exclusion chromatography (SEC) was conducted in 1,1,1,3,3,3-Hexafluoro-2-propanol HFiP using a PSS SECurity GPC system equipped with a PFG 7 µm 8 × 50 mm pre-column, a PSS 100 Å, 7 µm 8 × 300 mm and a PSS 1000 Å, 7 µm 8 × 300 mm column in series and a differential refractive index (RI) detector at a flow rate of 1.0 mL∙min^−1^. The system was calibrated against Agilent Easi-Vial linear poly (methyl methacrylate) (PMMA) standards and analyzed by the software package PSS winGPC UniChrom. The concentration of the SEC samples was 3 mg/mL HFiP. The dialysis purifications were performed using Snakeskin dialysis tubing (ThermoFisher, Waltham, MA, USA) with MWCO 3500 Da suspended in 2 L of deionized water. Rheological measurements were conducted using an MCR 301 digital rheometer (Anton Paar™, Physica, Australia). All experiments were completed at room temperature using a conical plate (CP50-1, Anton Paar™, Physica, Australia) consisting of a 50 mm diameter geometry and a gap length of 0.097 mm with the use of a protective hood to prevent evaporation. The morphology of the hydrogels was probed with a field emission scanning electron microscope (Zeiss Ultra SEM, Oberkochen, Germany). SEM analysis was conducted at the CRANN-Advanced Microscopy Laboratory (Dublin, Ireland). For the 3D extrusion printing, the hydrogels were loaded into the printing cartridge (Cellink™, Gothenburg, Sweden) using a spatula. The cartridge was then connected to the air system and placed in the printhead. The 3D printing was conducted at room temperature, using a Cellink™ INKREDIBLE+ 3D bioprinter operated at printing pressure ~160 kPa and printing speed ~70 mm/min. The nozzle diameter was 23G. The hydrogel inks were printed in pyramid architecture without further modification.

Synthesis of ε-carbobenzyloxy-L-Lysine (Lys(ZLL)) NCA. *ε*-Carbobenzyloxy-L-lysine (15 g, 53.51 mmol) and α-pinene (18.22 g, 133.78 mmol) were suspended in 180 mL of dry THF and heated under reflux. A solution of triphosgene (7.15 g, 24.08 mmol) in 30 mL dry THF was added dropwise to the suspension. The suspension was refluxed until all solids disappeared and the solution became clear. The solution was then cooled and filtered, and 2/3 of the volume was removed under vacuum. It was then precipitated by the addition of 250 mL hexane and stored overnight at −20 °C. The NCA solid was dried, redissolved in dry ethyl acetate and filtered. The NCA solution was then recrystallized thrice in ethyl acetate/hexane (1:3) and subsequently washed with hexane to remove any impurities. It was vacuum dried to afford a colorless fluffy solid (yield 80%).

Synthesis of O-benzyl-L-Tyrosine (BLT) NCA. *O*-benzyl-*L*-tyrosine (8 g, 29.49 mmol) and α-pinene (8.83 g, 64.87 mmol) were suspended in 110 mL of dry THF and heated under reflux. A solution of triphosgene (4.37 g, 14.74 mmol) in 20 mL dry THF was added dropwise to the suspension. The suspension was refluxed until all solids disappeared and the solution became clear. The solution was then cooled, filtered and reduced to 1/3 of its original volume under vacuum. It was then precipitated by the addition of 150 mL hexane and stored overnight at −20 °C. The NCA solid was dried, redissolved in dry ethyl acetate and reprecipitated in an excess of hexane. The NCA was then recrystallized twice in a mixture of ethyl acetate/hexane followed by vacuum drying to afford off-white colorless crystals (yield 79%).

Synthesis of S-benzyl-L-cysteine (Cys(Bz)) NCA. *S*-benzyl-*L*-cysteine (11 g, 52.06 mmol) and α-pinene (15.60 g, 114.54 mmol) were suspended in 130 mL of dry THF and heated under reflux. A solution of triphosgene (6.64 g, 22.39 mmol) in 25 mL dry THF was added dropwise to the suspension. The suspension was refluxed until all solids disappeared and the solution became clear. The solution was then cooled, filtered and reduced to 1/3 of its original volume under vacuum. It was then precipitated by the addition of 180 mL hexane and stored overnight at −18 °C. The NCA solid was dried, redissolved in dry ethyl acetate, reprecipitated in an excess of hexane and allowed to crystallize at −20 °C. The NCA was then recrystallized twice in a mixture of ethyl acetate/hexane followed by vacuum drying to afford shiny long crystals (yield 88%).

Synthesis of star copolypeptides. The following process is representative of the synthesis of 8-arm star PPI-p(Lys-*co*-Tyr-*b*-Cys(Bz)) with 400 monomeric units of lysine, 80 monomeric units of tyrosine and 80 monomeric units of cysteine (P3). A mixture of the NCA of ε-carbobenzyloxy-L-lysine (2.44 g, 0.0079 mol) and the NCA of O-benzyl-L-tyrosine (0.47 g, 0.0015 mol) was dissolved in 40 mL of anhydrous CHCl_3_ under an N_2_ atmosphere in a Schleck flask and placed in a thermostatically controlled bath (0 °C). A solution of G2 PPI dendrimer (0.0155 g, 0.019 mmol) in 1 mL CHCl_3_ was added to the dissolved NCAs solution via syringe. The solution was stirred at 0 °C overnight and periodically degassed under vacuum. FT-IR was used to monitor the complete consumption of the monomer. An aliquot was then taken directly via syringe to monitor the molecular mass using size exclusion chromatography (SEC). The NCA of S-benzyl-L-cysteine (0.37 g, 0.0015 mol) was then dissolved in 10 mL of anhydrous CHCl_3_ and charged to the flask. Stirring was continued until complete consumption was confirmed by FTIR. The polymer was then precipitated into an excess of diethyl ether and centrifuged for 5 min at 7000 revolutions per minute (rpm). The supernatant was decanted, and the solid polymer was dried in a vacuum oven at 37 °C overnight. The obtained polymer (2.5 g) was solubilized in 35 mL of trifluoroacetic acid and an excess of HBr (33 wt.% in acetic acid, 15 mL) and then added dropwise to the polymer solution and allowed to stir for 18 h. The polymer was precipitated into excess diethyl ether, and the supernatant decanted and washed three more times with diethyl ether. It was then dried under vacuum and subsequently dissolved in deionized water. Extensive dialysis was performed against deionized water for 5 days using a 10,000 MWCO membrane (Thermo Scientific™, Waltham, MA, USA), with frequent water replacement. Lyophilization then afforded the P3 polymer (1.2 g).

In vitro toxicity. Rat mesenchymal stem cells (rMSCs) were isolated from 6-8 week old Sprague Dawley rats as approved by the RCSI Research Ethics Committee using methods previously described [43]. Briefly, the femora of both hind limbs of the rats were clipped to expose the bone marrow, which was flushed out using expansion media using an 18 G needle and 12 mL syringe into a 65 mm Petri dish. The isolate was cultured in 15 mL of expansion media under standard conditions of 37 °C, 5% CO_2_, and 95% relative humidity for 24 h to allow for the formation of adherent colonies. The cells were tested for mycoplasma at P1 and underwent tri-lineage differentiation at P4. All experiments were carried out with cells between P4 and P6. Rat mesenchymal stem cells (rMSCs) were cultured in T175 flasks at 37 °C and 5% CO_2_. rMSCs were maintained with Dubbeco’s modified high glucose media (Sigma, Dublin, Ireland) containing supplements of 20% fetal bovine serum (Sigma, Taufkirchen, Germany), 1% GLUTmax (Gibco, Dublin, Ireland), 1% non-essential amino acids (Gibco, Ireland) and 0.2% primocin (InvivoGen, Toulouse, France). For biocompatibility testing, the rMSCs were plated at a density of 2 × 10^4^ cells per well in an adherent 24-well plate. A total volume of 220 μg of the hydrogel was placed in a Corning Transwell^®^ cell culture insert with a pore size of 4 μm. The insert was placed in the well with the rMSCs. After 1 day, 3 days and 7 days of culture, the Transwell^®^ insert containing the hydrogel was removed from the well, as was the medium. The rMSCs were washed with PBS and a 10% solution of alamarBlue© in medium was added to each well and allowed to incubate for 1 h at 37 °C, 5% CO_2_. Fluorescence was then measured on a Tecan plate reader with an excitation wavelength of 545 nm and an emission wavelength of 590 nm.

Additionally, LIVE/DEAD™ (Invitrogen™, Waltham, MA, USA) staining was carried out to further assess cytocompatibility and observe cellular morphology. The medium was removed from all the wells and the cells were rinsed with PBS. 200 µL of LIVE/DEAD™ solution (as per the manufacturer’s protocol) was added and left to incubate for 5 min protected from light at room temperature. Wells were imaged using a fluorescent microscope (Leica Microsystems, Wetzlar, Germany) and the resultant live and dead images were merged using ImageJ software version 2.0.

Bactericidal Assays. P3 polymer (1.65 mg) was weighed in microcentrifuge tubes (Eppendorf, Hamburg, Germany) and sterile water (163.35 μL) was added to form hydrogels within the tubes. *E. coli* strain ATCC25922 and *S. aureus* strain ATCC25923 were grown overnight at 37 °C on Mueller Hinton (MH) agar plates. Single colonies were used to prepare suspensions in sterile PBS to the density of a 0.5 McFarland standard (Approximately 10^7^ colony-forming units (CFU)/mL) using a Densichek^TM^ meter (Biomerieux, Dublin, Ireland). Suspensions were further diluted 1/100 in PBS to approximately 10^5^ CFU/mL. Aliquots of these diluted suspensions were added to the hydrogels. Assays were incubated for 40 min at room temperature. Control series were included in which the diluted suspensions were incubated without hydrogels. Samples were removed from the hydrogel surface and serially diluted in sterile PBS; 100 μL aliquots from these dilutions were spread onto MH agar plates, incubated at 37 °C overnight and colonies were counted the following day. CFU/mL (surviving bacteria) and the log reduction in CFU/mL relative to controls were calculated.

## Figures and Tables

**Table 1:** Molecular characteristics of the synthesized star block copolypeptides.

Sample	Polymer	DP ^a)^ Lys ^theor.^	DP ^a)^ Tyr ^theor.^	DP ^a)^ Cys(Bz) ^theor.^	*M_n_*^b)^ 1st Block [kg mol^−1^]	*M_n_*^b)^ 2nd Block [kg mol^−1^]	*Ð* ^c)^
P1	PPI-p(Tyr-*b*-Lys-*b*-Cys(Bz))	400	80	80	24	106	1.27
P2	PPI-p(Lys-*b*-Tyr-*b*-Cys(Bz))	400	80	80	84	104	1.28
P3	PPI-p(Lys-*co*-Tyr-*b*-Cys(Bz))	400	80	80	74	-	1.16

^a)^ Theoretical degree of polymerization. ^b)^ Protected star block copolypeptides; obtained by SEC in HFIP using PMMA standards. ^c)^ Polydispersity indices of the protected second block for P1 and P2 and the protected statistical inner block for P3 obtained by SEC.

**Table 2:** Hydrogel properties.

Sample	Maximum Ratio of Polymer Mass/Hydrogel Mass ^a)^	G′ (Pa) ^b)^	Tan(delta) ^b)^
P1	1/15	18,119	0.109
P2	1/33	2796	0.142
P3	1/100	5439	0.092

^a)^ Vial inversion was used to obtain the point of maximum water uptake while still having hydrogel properties. ^b)^ Obtained by rheology measurements at 25 °C. G′ = Storage modulus, G″ = Loss factor, Tan(delta)= G″/G′.

## Data Availability

The original contributions presented in the study are included in the article/Supplementary Material, further inquiries can be directed to the corresponding author.

## Supplementary Materials

The following supporting information can be downloaded at: https://www.mdpi.com/article/10.3390/gels10100652/s1: Figure S1: Synthesis of N(ε)-benzyloxycarbonyl-L-lysine NCA; Figure S2: FTIR spectrum of N(ε)-benzyloxycarbonyl-L-lysine NCA; Figure S3: ^1^H-NMR spectrum of N(ε)-benzyloxycarbonyl-L-lysine NCA. (DMSO-d6); Figure S4: Synthesis of O-benzyl-L-Tyrosine NCA; Figure S5: FTIR spectrum of O-benzyl-L-Tyrosine NCA; Figure S6: ^1^H-NMR spectrum of O-benzyl-L-Tyrosine NCA. (DMSO-d6); Figure S7: Synthesis of S-benzyl-L-cysteine NCA; Figure S8: FTIR spectrum of S-benzyl-L-cysteine NCA; Figure S9: ^1^H-NMR spectrum of S-benzyl-L-cysteine NCA. (DMSO-d6); Figure S10: FTIR spectrum of PPI-P(Lys-*co*-Tyr-*b*-Cyst(Bz)); Figure S11: ^1^H-NMR spectrum of P2. (DMSO-d6); Figure S12: ^1^H-NMR spectrum of P3 (DMSO-d6); Figure S13: Photo of PPI-P(Lys(Z)-*co*-Tyr(Bz)-*b*-Cyst(Bz)) in HFiP. The polymer is not soluble in the solvent; Figure S14: SEC eluogram of the inner blocks of P1 in HFiP before deprotection; Figure S15: SEC eluogram of the inner blocks of P2 in HFiP before deprotection; Figure S16: SEC eluogram of the inner block of P3 in HFiP before deprotection; Figure S17: Loss factor tan(δ) = G″/G′ of the presented hydrogels; Figure S18: Frequency-dependent storage G′ and loss G″ moduli for the hydrogels P3 at different ratios of polymer mass/hydrogel mass; Figure S19: CD spectrum of P3; Figure S20: FTIR spectrum of P3; Figure S21: SEM picture of P1; Figure S22: SEM picture of P2; Table S1: Extrudability of hydrogels through different needles gauge at maximum ratio of polymer mass/hydrogel mass.
