# Supplementary material for: Shear-Thinning Extrudable Hydrogels Based on Star Polypeptides with Antimicrobial Properties
Source: Gels. 2024 Oct 11;10(10):652. doi: 10.3390/gels10100652 (PMC11507159; doi:10.3390/gels10100652)
Supplement: Supplementary file 1 [file gels-10-00652-s001.zip › gels-3234569-supplementary.pdf]

Supplementary material of:

Shear thinning extrudable hydrogels based on star polypeptides with antimicrobial properties

Dimitrios Skoulas [1*], Muireann Fallon[2], Katelyn Genoud[3], Fergal J. O'Brien[3,4,5], Deirdre Fitzgerald Hughes[2] , and Andreas Heise [1,4,5]

1 Department of Chemistry, RCSI University of Medicine and Health Sciences, 123 St. Stephen's Green, D02 YN77 Dublin, Ireland.; andreasheise@rcsi.ie

2 Department of Clinical Microbiology, Royal College of Surgeons in Ireland, RCSI Education and Research, Beaumont Hospital, Beaumont, Dublin, Ireland.

3 Tissue Engineering Research Group, Department of Anatomy and Regenerative Medicine, RCSI, Dublin D02 YN77, Ireland.

4 Science Foundation Ireland (SFI) Centre for Research in Medical Devices (CURAM), D02 YN77 Dublin, Ireland.

5 AMBER, The SFI Advanced Materials and Bioengineering Research Centre, D02 YN77 Dublin, Ireland.

* Correspondence: dskoulas@hotmail.gr

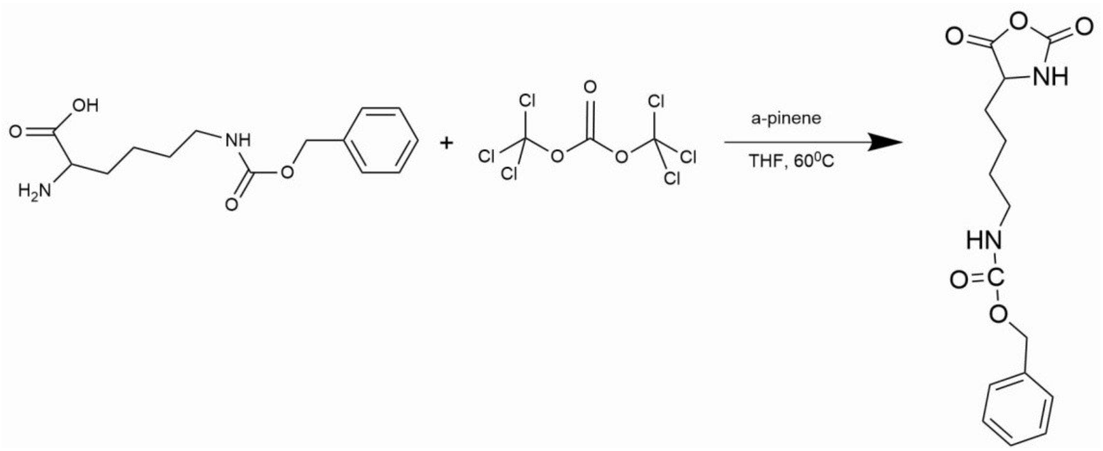

Figure S1: Synthesis of N(ε)-benzyloxycarbonyl-L-lysine NCA.

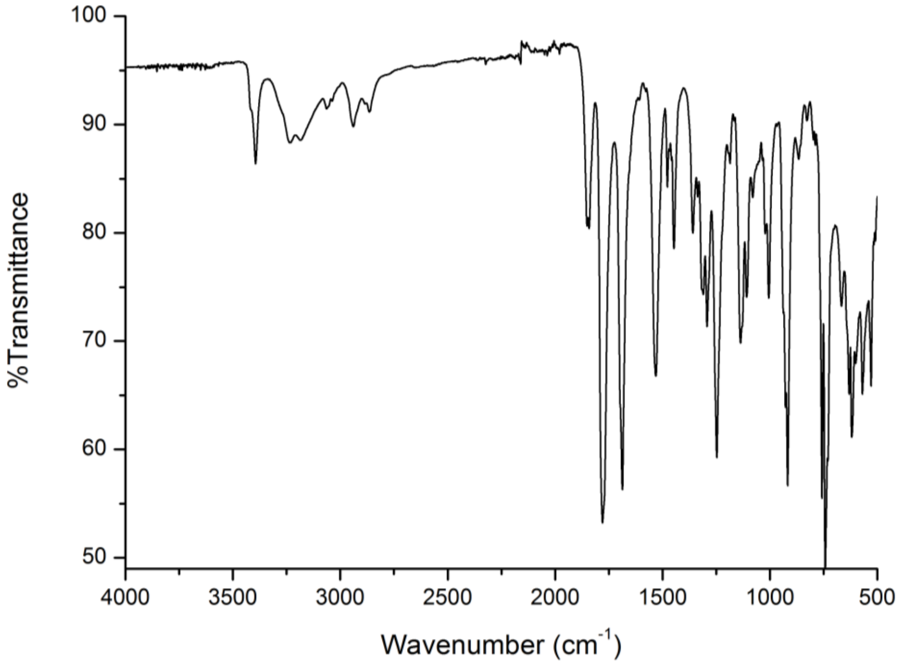

Figure S2: FTIR spectrum of N(ε)-benzyloxycarbonyl-L-lysine NCA.

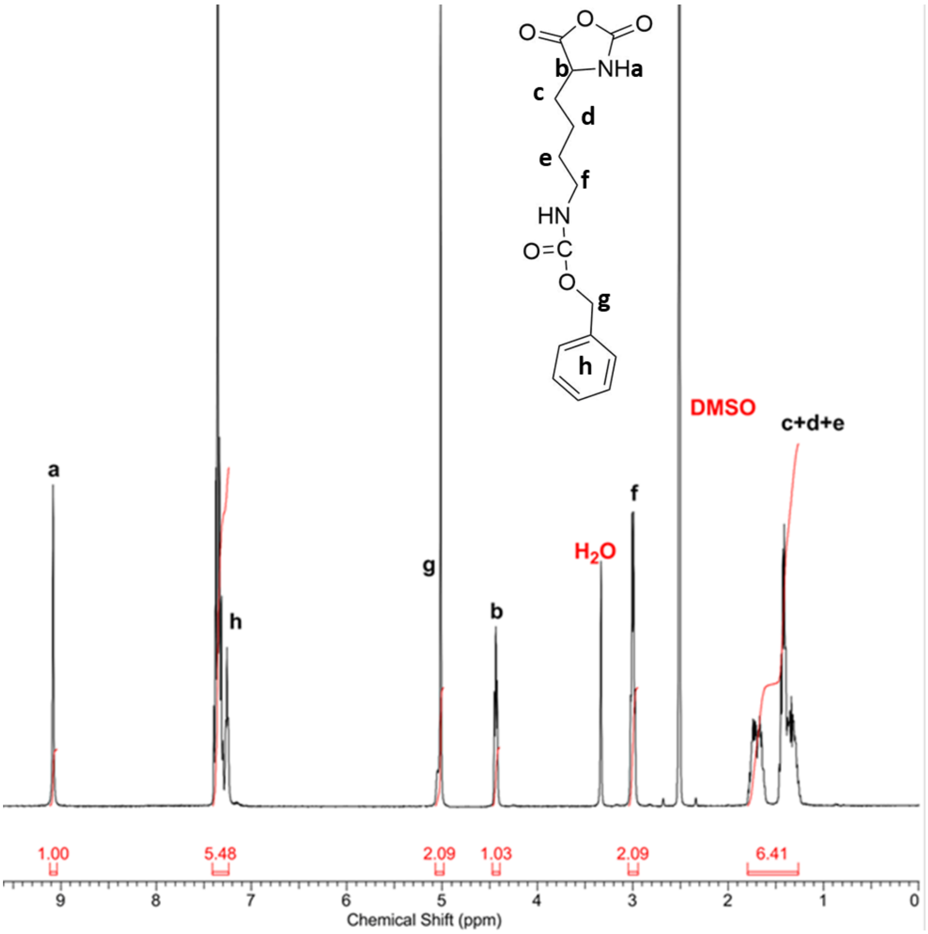

Figure S3: ^{1}H-NMR spectrum of N(ε)-benzyloxycarbonyl-L-lysine NCA. (DMSO-d6).

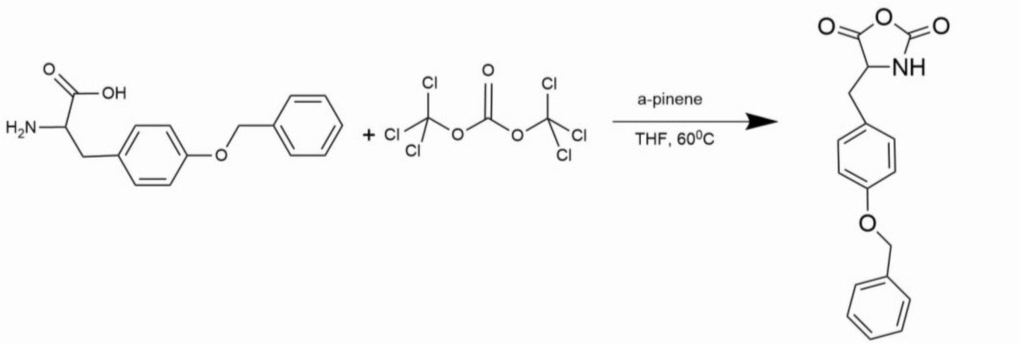

Figure S4: Synthesis of O-benzyl-L-Tyrosine NCA.

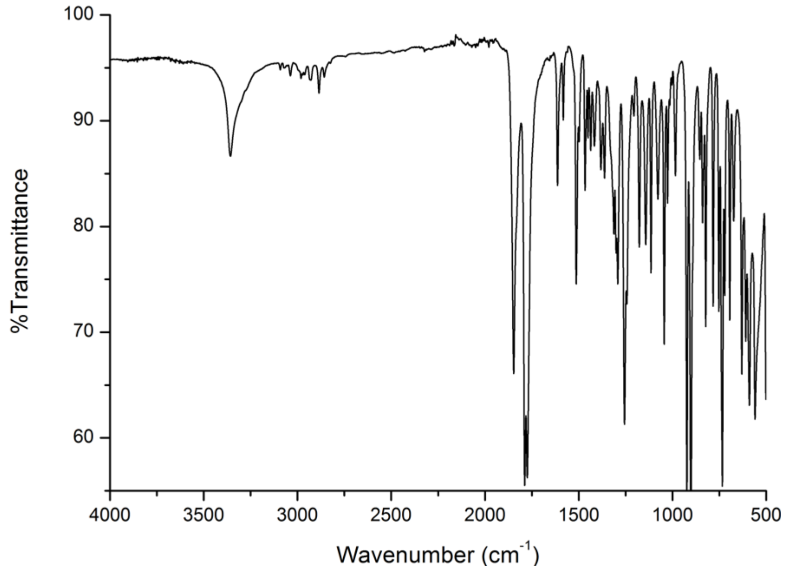

Figure S5: FTIR spectrum of O-benzyl-L-Tyrosine NCA.

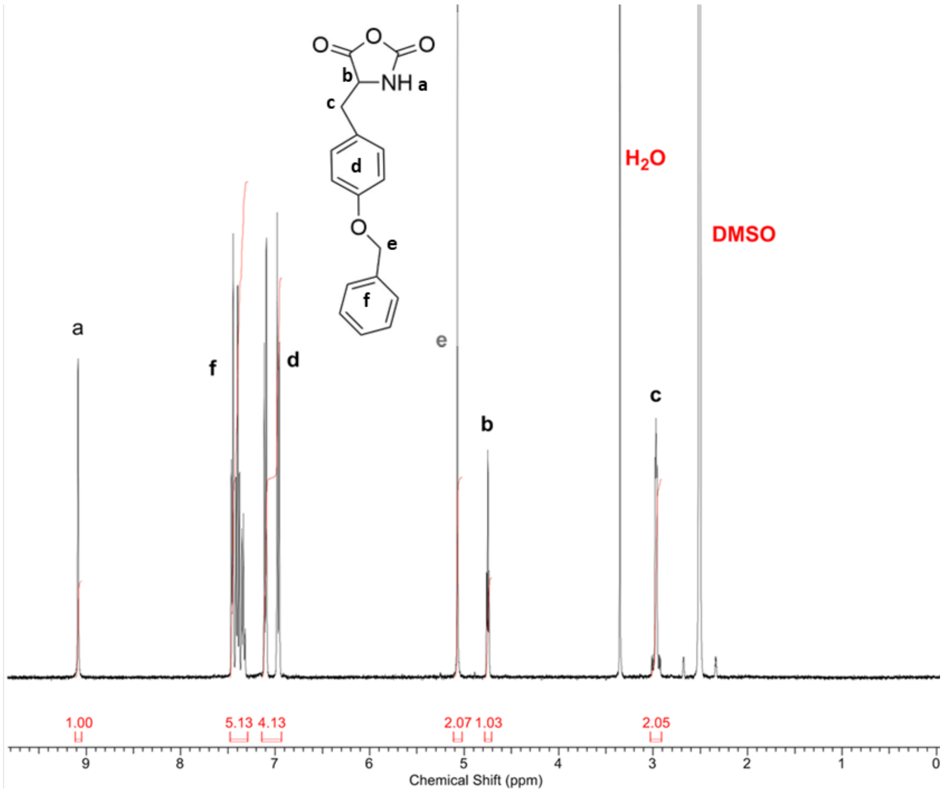

Figure S6: ^{1}H-NMR spectrum of O-benzyl-L-Tyrosine NCA. (DMSO-d6).

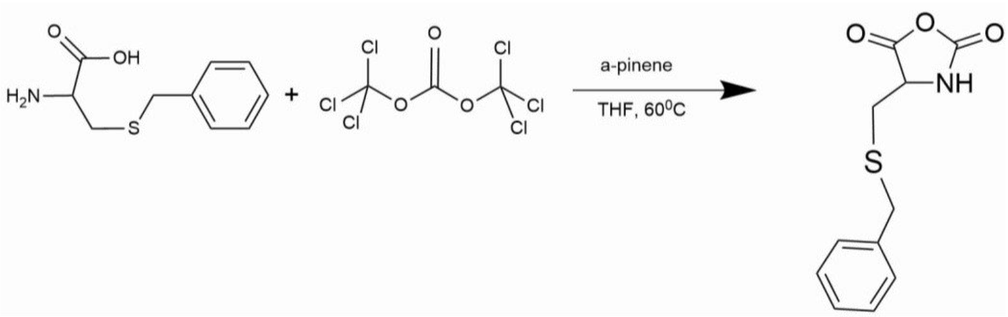

Figure S7: Synthesis of S-benzyl-L-cysteine NCA.

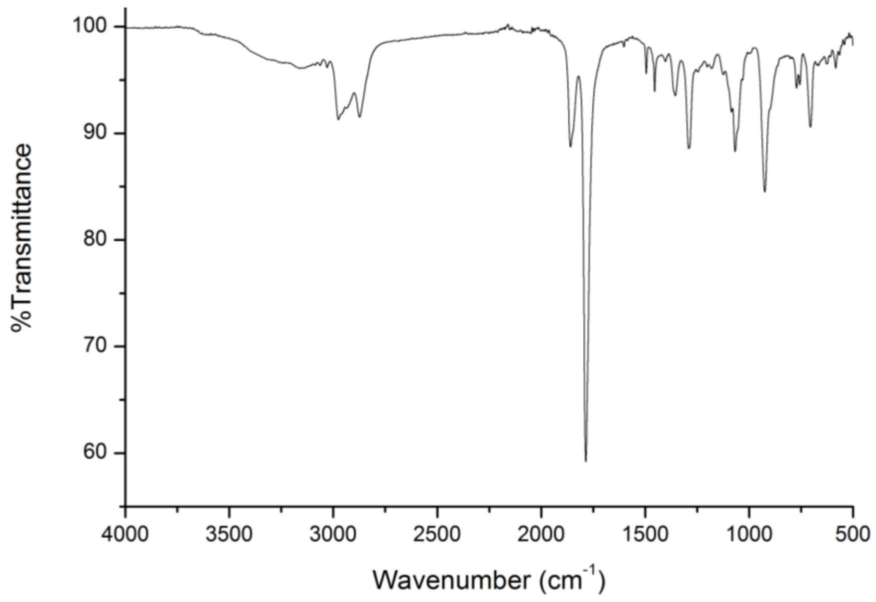

Figure S8: FTIR spectrum of S-benzyl-L-cysteine NCA.

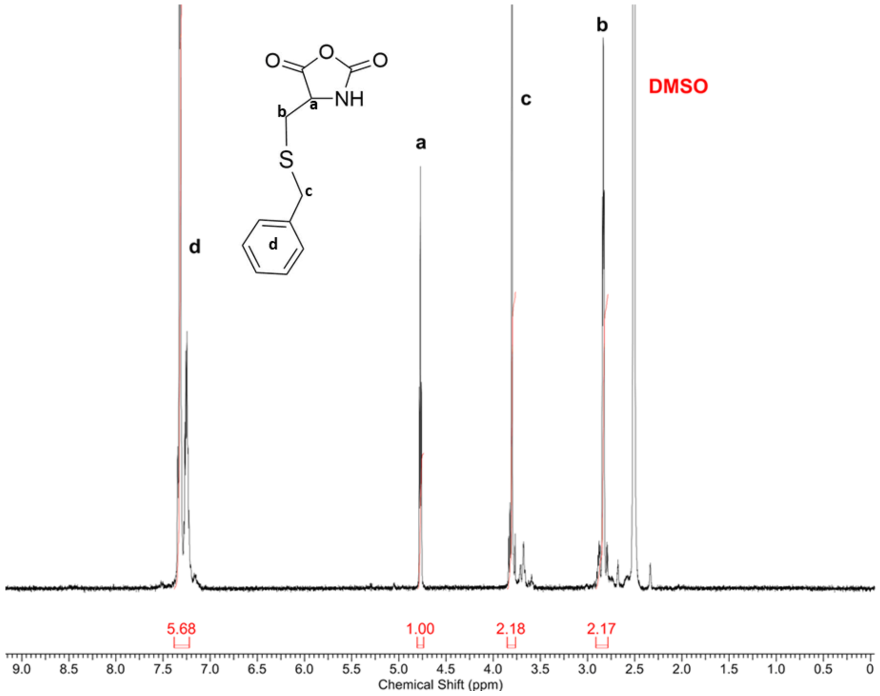

Figure S9: ^{1}H-NMR spectrum of S-benzyl-L-cysteine NCA. (DMSO-d6).

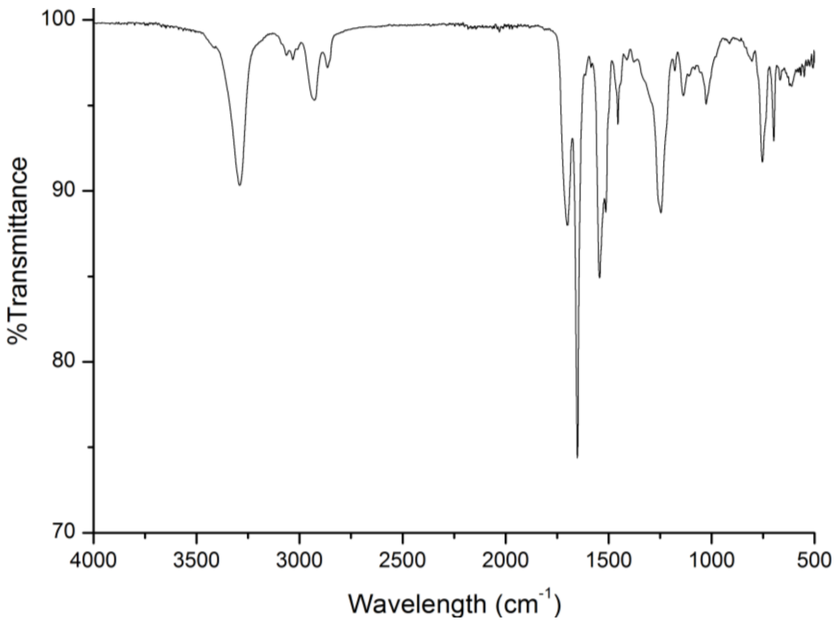

Figure S10: FTIR spectrum of PPI-P(Lys-*co*-Tyr-*b*-Cyst(Bz))

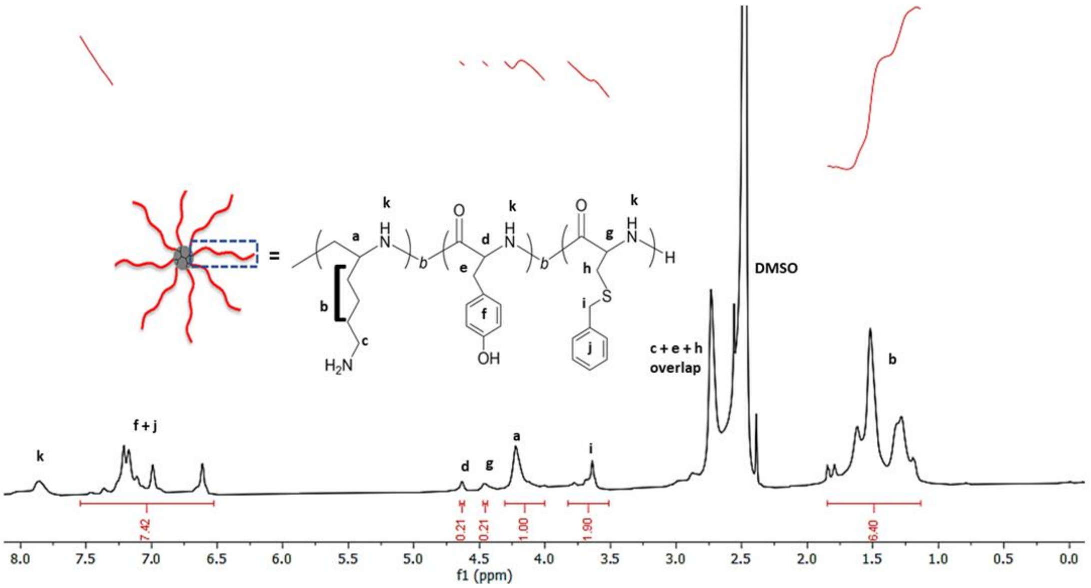

Figure S11: ^{1}H-NMR spectrum of P2. (DMSO-d6).

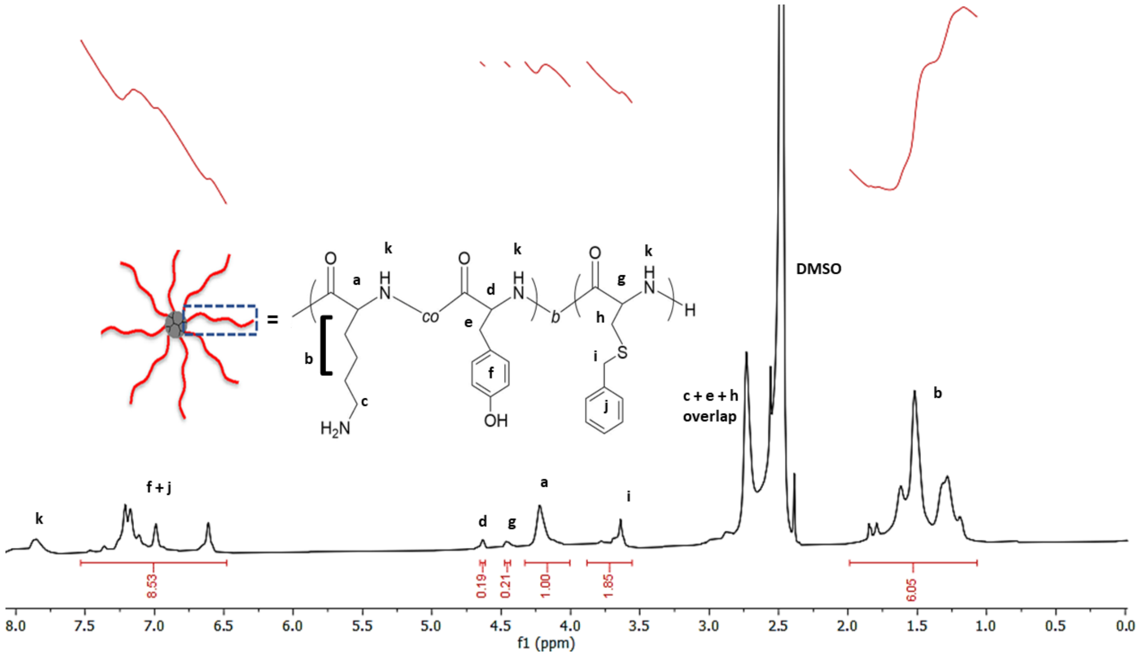

Figure S12: ^{1}H-NMR spectrum of P3 (DMSO-d6).

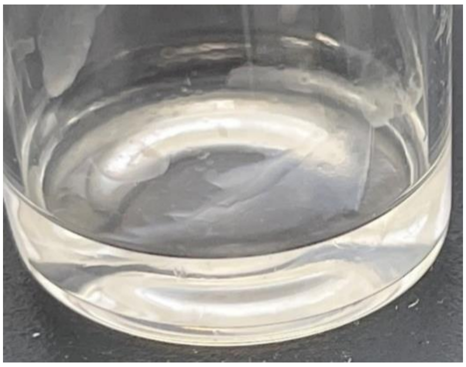

Figure S13: Photo of PPI-P(Lys(Z)-*co*-Tyr(Bz)-*b*-Cyst(Bz)) in HFiP. The polymer is not soluble in the solvent.

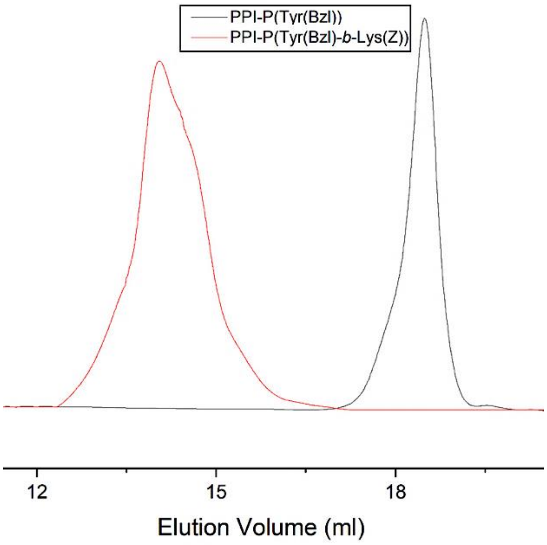

Figure S14: SEC eluogram of the inner blocks of P1 in HFiP before deprotection.

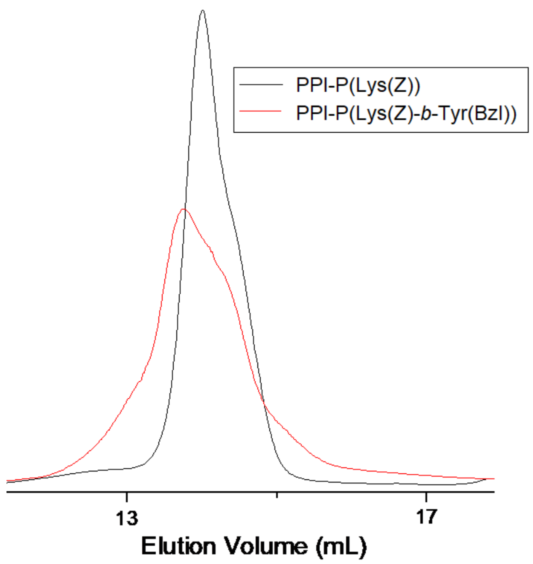

Figure S15: SEC eluogram of the inner blocks of P2 in HFiP before deprotection.

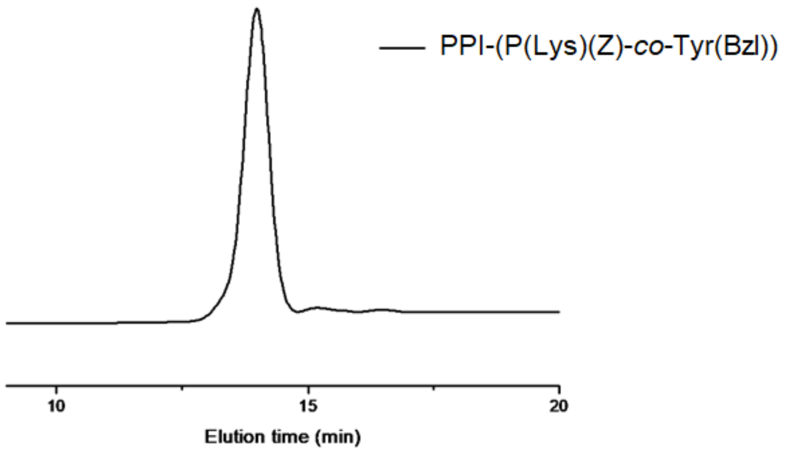

Figure S16: SEC eluogram of the inner block of P3 in HFiP before deprotection.

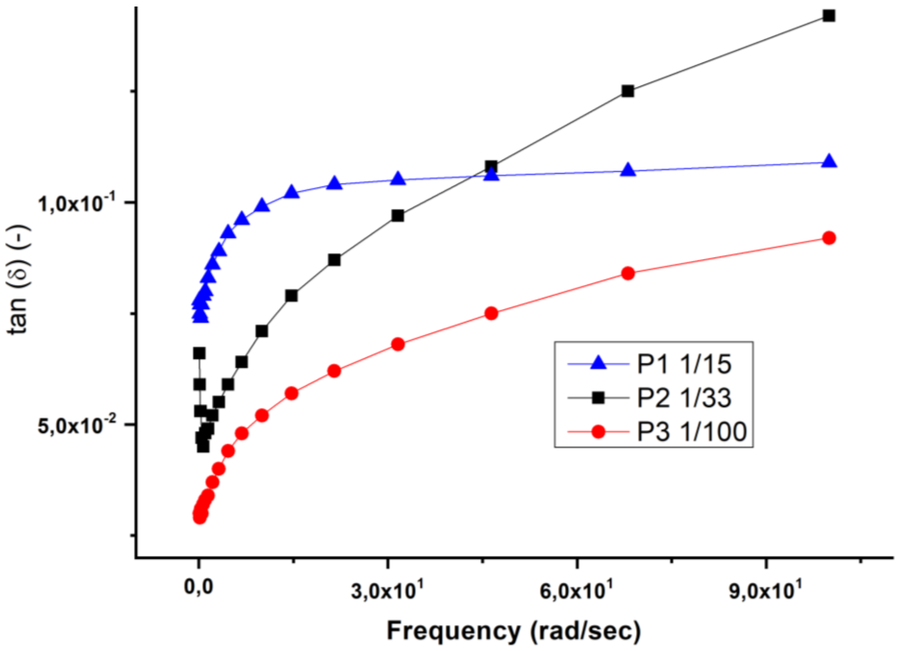

Figure S17: Loss factor tan(δ) = G″/G′ of the presented hydrogels.

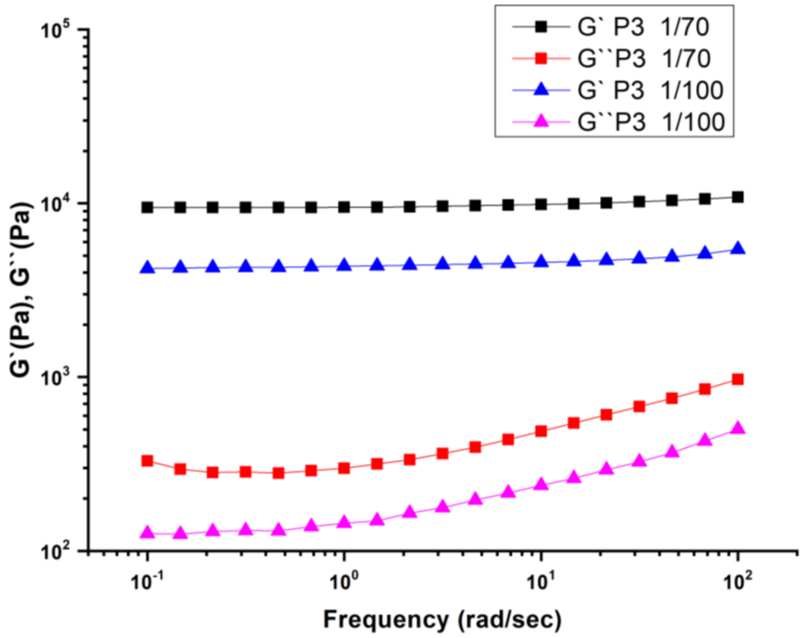

Figure S18: Frequency-dependent storage G′ and loss G″ moduli for the hydrogels P3 at different ratio of polymer mass/ hydrogel mass.

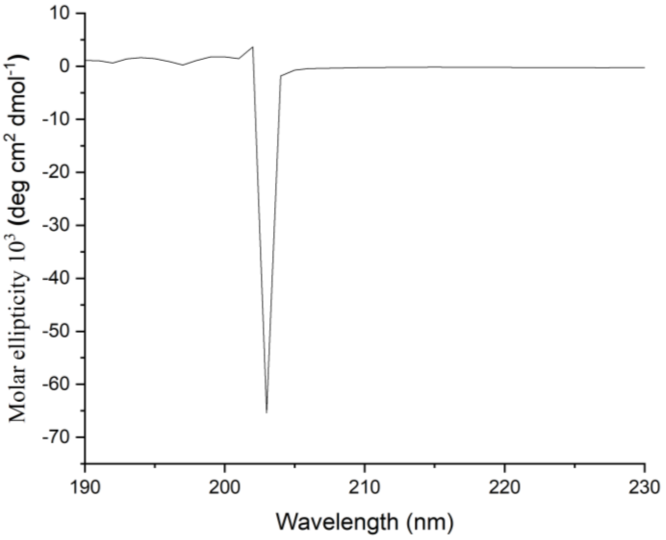

Figure S19: CD spectrum of P3.

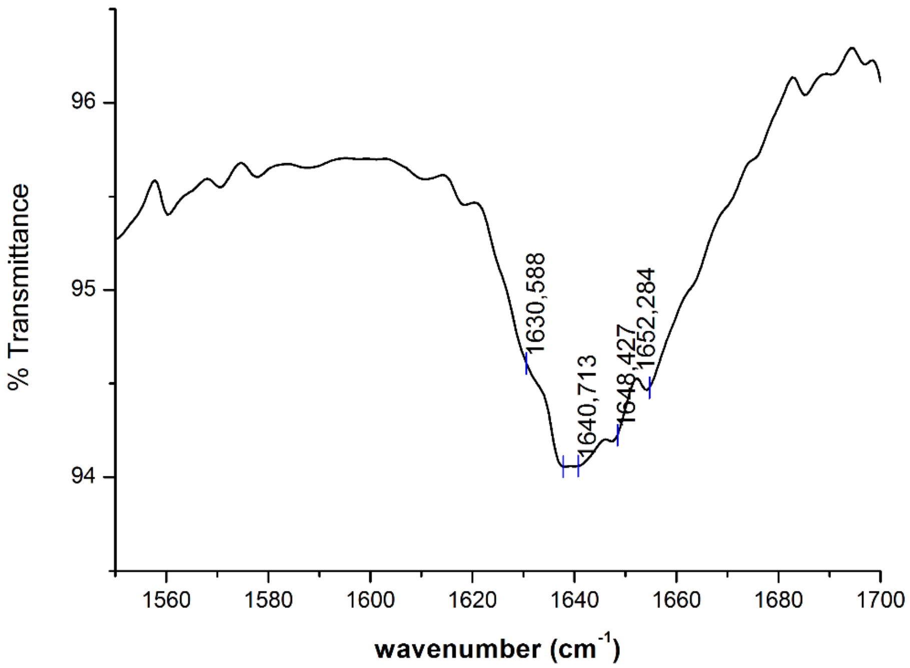

Figure S20: FTIR spectrum of P3.

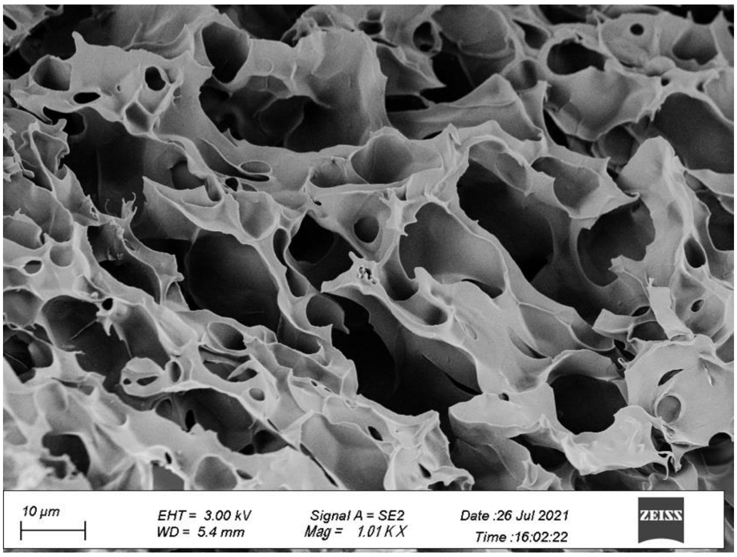

Figure S21: SEM picture of P1.

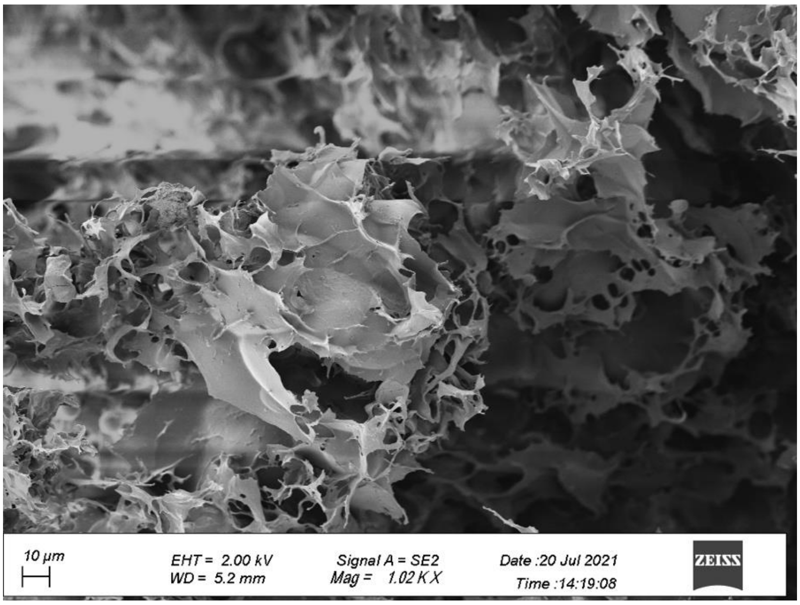

Figure S22: SEM picture of P2.

Table S1. Extrudability of hydrogels through different needles gauge at maximum ratio of polymer mass/ hydrogel mass.

	Needle gauge								
Sample	14	16	18	20	21	22	23	25	27
P1	✓	✓	✓	✗	✗	✗	✗	✗	✗
P2	✓	✓	✓	✗	✗	✗	✗	✗	✗
P3	✓	✓	✓	✓	✓	✓	✓	✓	✓
